# Real-time *in vivo* optical imaging of glioma with confocal laser endomicroscopy in a murine model: comparative analysis of performance of indocyanine green and fluorescein sodium

**DOI:** 10.3389/fonc.2026.1906912

**Published:** 2026-09-09

**Authors:** Yuan Xu, Yoon Hwan Byun, Shin-Hyuk Kang, Mark C. Preul

**Affiliations:** 1 The Loyal and Edith Davis Neurosurgical Laboratory, Department of Neurosurgery, Barrow Neurological Institute, St. Joseph’s Hospital and Medical Center, Phoenix, AZ, United States; 2 Department of Neurosurgery, SMG-SNU Boramae Medical Center, Seoul National University College of Medicine, Seoul, Republic of Korea; 3 Department of Neurosurgery, Korea University Anam Hospital, Korea University College of Medicine, Seoul, Republic of Korea

**Keywords:** brain tumor, confocal laser endomicroscopy, fluorescein sodium, fluorescence-guided surgery, glioma, indocyanine green, intraoperative imaging, optical biopsy

## Abstract

**Objective:**

Confocal laser endomicroscopy (CLE) enables real-time, cellular-resolution optical biopsies during surgery. Although fluorescein sodium (FNa)–based CLE has been studied extensively, few studies have examined near-infrared (NIR) CLE imaging using indocyanine green (ICG) in brain tumor models. This study compared NIR CLE imaging using intravenous or topical ICG with blue laser CLE using FNa in a mouse glioma model.

**Methods:**

Mice with orthotopically implanted GL261 glioma underwent bilateral craniectomy and CLE imaging. Four mice received intravenous ICG, and 4 received topical ICG. Two mice each received intraperitoneal or topical FNa. CLE imaging was performed using the NIR CLE system and the FNa CLE system on tumor, contralateral normal cortex, and postresection margins and correlated with hematoxylin and eosin (H&E)–stained sections. Signal-to-background ratios (SBRs) were analyzed using an automated 3-step segmentation pipeline and compared between intravenous and topical ICG groups.

**Results:**

NIR CLE with intravenous or topical ICG produced a characteristic intracellular fluorescence pattern in tumor tissue, with strong cytoplasmic fluorescence surrounding dark, round nuclei, enabling visualization of cell size, morphology, and pleomorphism consistent with H&E-stained sections. Tumor cells at resection margins were reliably identified by either method. Topical ICG staining of contralateral normal cortex revealed distinct features consistent with normal brain tissue and clearly distinguishable from tumor. Systemic FNa produced a negative-contrast extracellular pattern with tumor cells that appeared as dark silhouettes on a bright background, whereas topical FNa stained tumor and normal tissue with limited morphological differentiation. SBR analysis of intravenous and topical ICG images showed topical ICG produced significantly higher SBRs than intravenous ICG at image level (median 5.7 vs. 4.9, p<0.001) but not animal level.

**Conclusions:**

NIR CLE using ICG provided high-quality cellular-resolution imaging of glioma and normal brain tissue. ICG enabled visualization of cell size and morphology, facilitating clear differentiation between tumor and normal brain. Topical ICG application produced an imaging pattern equivalent to intravenous administration with a similar SBR and allowed visualization of normal brain cytoarchitecture. These findings support ICG for intraoperative NIR CLE imaging of brain tumors and provide a foundation for investigating topical ICG use with CLE imaging.

## 1 Introduction

Confocal laser endomicroscopy (CLE) is a miniaturized optical imaging technology that provides real-time, *in vivo*, cellular-resolution fluorescence imaging, enabling “optical biopsies” of tissue without the need for specimen removal and processing (1). During neurosurgery, the hand-held probe is applied directly to the tissue surface, permitting visualization of cytoarchitectural features, including hypercellularity, cellular pleomorphism, abnormal vasculature, and necrosis, characteristic of tumor versus normal brain tissue (1–5).

Two CLE systems are cleared for clinical neurosurgical applications: Convivo (Carl Zeiss Meditec AG) and cCeLL (VPIX Medical, Inc). The Convivo system operates a 488-nm blue laser that uses fluorescein sodium (FNa) as the fluorescent contrast agent. The cCeLL system uses a 775-nm near-infrared (NIR) laser optimized for indocyanine green (ICG) contrast (6).

FNa does not exhibit tumor cell–specific uptake (7–9). When administered intravenously, FNa distributes into areas of blood-brain barrier (BBB) disruption, creating an extracellular fluorescence pattern that delineates tumor cells as dark contours on a bright background on CLE images (10, 11). ICG is widely used as a fluorophore for rapid intraoperative neurosurgical fluorescence angiography. Like FNa, ICG selectively accumulates in tissue with a disrupted BBB, leading to the investigation of ICG for brain tumor surgery guidance with NIR camera devices (12). ICG administration using the second-window technique was developed to leverage the enhanced permeability and retention effect to delineate brain tumor tissue (13). Use of ICG with CLE for brain tumor imaging was first reported in 2011 using a preclinical NIR CLE system, demonstrating that CLE imaging with ICG could provide real-time histological information and enhance the sensitivity of tumor detection because of its selectivity for tumor cells (14). Recently, several studies have demonstrated the clinical feasibility of cCeLL for intraoperative brain tumor diagnosis, with CLE images effectively visualizing tumor cytoarchitecture (6, 15).

To our knowledge, no study has compared the 2 clinically available CLE imaging systems in a controlled setting. In this study, we compared CLE imaging with 2 fluorophores under 2 different administration conditions, namely intravenous and topical, using the 2 CLE systems. We evaluated imaging characteristics, tumor-to-background ratios, and histological correlations. The results of this study provide the first demonstration of both CLE systems side by side in a single clinically relevant experimental tumor model. We establish how the choice of fluorophore and delivery route affects the ability to delineate brain tumors microscopically *in vivo* and to detect remnant tumor cells at the resection margin.

## 2 Materials and methods

### 2.1 Animal model and tumor implantation

Experimental procedures were approved by the Institutional Animal Care and Use Committee of St. Joseph’s Hospital and Medical Center (Protocol No. 637). Eight- to 10-week-old female C57BL/6J mice (The Jackson Laboratory) with a mean body weight of approximately 25 g were used. Orthotopic implantation of GL261 mouse glioma cell line was performed according to previously established methods (16, 17). Two microliters of GL261 cells (1 × 10^7^ cells/mL) were injected into the right striatum slowly over 2 minutes. Tumor-bearing mice were operated on approximately 20 days after implantation.

### 2.2 Fluorophore administration

For intravenous ICG administration, 0.1 mg/mL ICG (IC-Green, Diagnostic Green LLC) was injected intravenously into the tail vein at a dose of 0.4 mg/kg, as described elsewhere (14). For systemic FNa administration, 5% FNa was injected intraperitoneally at a dose of 5 mg/kg body weight immediately before CLE imaging (17). For topical ICG and FNa application, 2 to 3 drops of 0.0001% ICG solution or 5% FNa was applied to the region of interest (ROI) and incubated for 1 minute. Tissue was irrigated profusely with normal saline to remove excessive fluorophore and minimize nonspecific background fluorescence.

### 2.3 Confocal laser endomicroscopy systems

Two CLE systems were used, each paired with its corresponding fluorophore. The cCeLL system was used for NIR CLE imaging with ICG. The system uses a dual laser channel configuration: a 775-nm laser with an 800–860-nm bandpass emission filter optimized for ICG fluorescence detection, and a 488-nm laser with a 510–560-nm bandpass filter used with FNa fluorescence (6). The cCeLL system uses a Lissajous scanning pattern that resonantly scans in both x and y axes, enabling fast scanning at a nonadjustable Z plane. The system provides a field of view of 500 × 500 μm^2^, with a lateral resolution of approximately 1.4 μm and an axial resolution of approximately 20 μm. It captures images at a constant rate of 10 frames per second. The ViewnVivo (Optiscan Imaging, Ltd) system, employing a 488-nm blue laser, was used with FNa. This system provided a field of view of 475 × 267 μm^2^ and a lateral resolution of 0.55 μm. The Z-depth is adjustable and ranges from 0 to 400 μm. Images are captured at frame rates up to 3.5 frames per second, with optimal image quality achieved at 0.8 frames per second. Probes for both systems were mounted in a 3-axis probe holder attached to an articulated arm for stable imaging (18).

### 2.4 Animal surgery and CLE imaging protocol

After animal anesthesia with a ketamine (80 mg/kg) and xylazine (10 mg/kg) cocktail, bilateral craniectomies were performed to expose both hemispheres. The fluorophore was then administered, and the imaging probe was carefully positioned in contact with the exposed tumor. CLE imaging was initiated with continuous capture, and the probe was moved across the cortex along the x- and y-axes to scan the tissue in one hemisphere, after which the cortex of the contralateral hemisphere was imaged. Macroscopically visible tumor tissue was removed with gentle, constant suction applied manually through a 5-mL syringe with a 20-gauge needle. Once adequate tumor removal was confirmed under the surgical microscope, the tumor resection bed was imaged with CLE. For animals that received topical fluorophores, the fluorophores were reapplied to the contralateral hemisphere and resection bed in the same fashion immediately before imaging. Throughout the imaging procedure, the laser power of both systems was adjusted to ensure an adequate fluorescence signal and maintain a low autobrightness level to avoid excessive image noise.

In a subset of animals, 0.01% topical acridine orange, a fluorophore with a spectrum similar to FNa that selectively stains cell nuclei, was co-administered with topical ICG, and the tumor was imaged sequentially using the cCeLL system’s 488-nm and 775-nm laser channels without repositioning the probe, permitting direct overlay of nuclear and cytoplasmic staining patterns in the same cells.

### 2.5 Histological processing and correlation

Following CLE imaging, the mice were euthanized via intraperitoneal injection of the ketamine and xylazine cocktail, administered at 5 times the standard anesthesia dose (ketamine 400 mg/kg, xylazine 50 mg/kg), and the brains were harvested. Whole brains were sectioned into coronal slices. Coronal sections containing the CLE imaging ROI were identified and fixed in 10% neutral buffered formalin and subsequently processed for standard paraffin embedding, sectioned into 5-µm slices, and stained with hematoxylin and eosin (H&E). To our knowledge, no previous study has reported an effect of FNa or ICG administration on subsequent H&E-stained histology (2, 4). H&E-stained slides were reviewed to confirm the presence and location of tumor tissue and to identify tumor cells at the margins. CLE images from each ROI were visually compared to the corresponding H&E-stained sections to assess correlation between CLE imaging findings and conventional histopathology. CLE images and corresponding H&E-stained sections were reviewed by a single investigator experienced in CLE imaging and GL261 mouse glioma histology (Y.X.).

### 2.6 Signal-to-background ratio analysis

Signal-to-background ratio (SBR) analysis was performed using Fiji software (National Institutes of Health) (19). After the image was converted to 8-bit grayscale, a 3-step segmentation pipeline was applied to define cytoplasm (signal), nuclei (excluded), and background regions. First, cell regions were identified using Li’s minimum cross-entropy thresholding method, which was selected for its ability to capture both bright and dim cytoplasm typically seen in CLE images (20). Morphological hole-filling ensured that enclosed nuclear regions remained within the cell boundary. Dark nuclear regions within cells were identified by applying Otsu’s threshold to the inverted intensity values of cell-interior pixels only to avoid confusion with extracellular dark regions (21). The resulting nuclei mask was refined by morphological erosion (radius = 2 pixels) to exclude cytoplasm bleed at the nuclei-cytoplasm boundary. The cytoplasm signal mask was then defined by subtracting the nuclei mask from the whole-cell mask. Background was defined by excluding the cytoplasm and nuclear pixels. The mean intensities of the cytoplasm and background pixels were calculated for each image. SBR was defined as the ratio of the mean cytoplasmic intensity to the mean background intensity.

### 2.7 Statistical analysis

Parametric data were presented as mean and standard deviation, and nonparametric data were presented as median and interquartile range. Because the data were not normally distributed, Mann-Whitney *U* tests were used to compare SBR, mean cytoplasmic fluorescence intensity, and mean background fluorescence intensity between the 2 groups. Because images from the same animal cannot be treated as fully independent observations, a linear mixed-effects model was fit using all available images to formally account for the clustering of images within animals while retaining the full dataset. Statistical significance was set at p < 0.05. Statistical analysis was performed using GraphPad Prism 10.4.0 (GraphPad Software).

## 3 Results

### 3.1 Animal and tumor characteristics

Four mice were used for CLE imaging with intravenous and topical ICG administration each. Two mice were imaged after intraperitoneal and topical FNa application each. All mice had adequate tumor growth extending from the right striatum superficially to just beneath the cortical surface. Exposure of the tumor was achieved with a small parallel incision on the right hemisphere cortex, approximately 2 mm lateral to the superior sagittal sinus, allowing for optimal CLE imaging.

### 3.2 Imaging with NIR CLE

**Figure 1:**
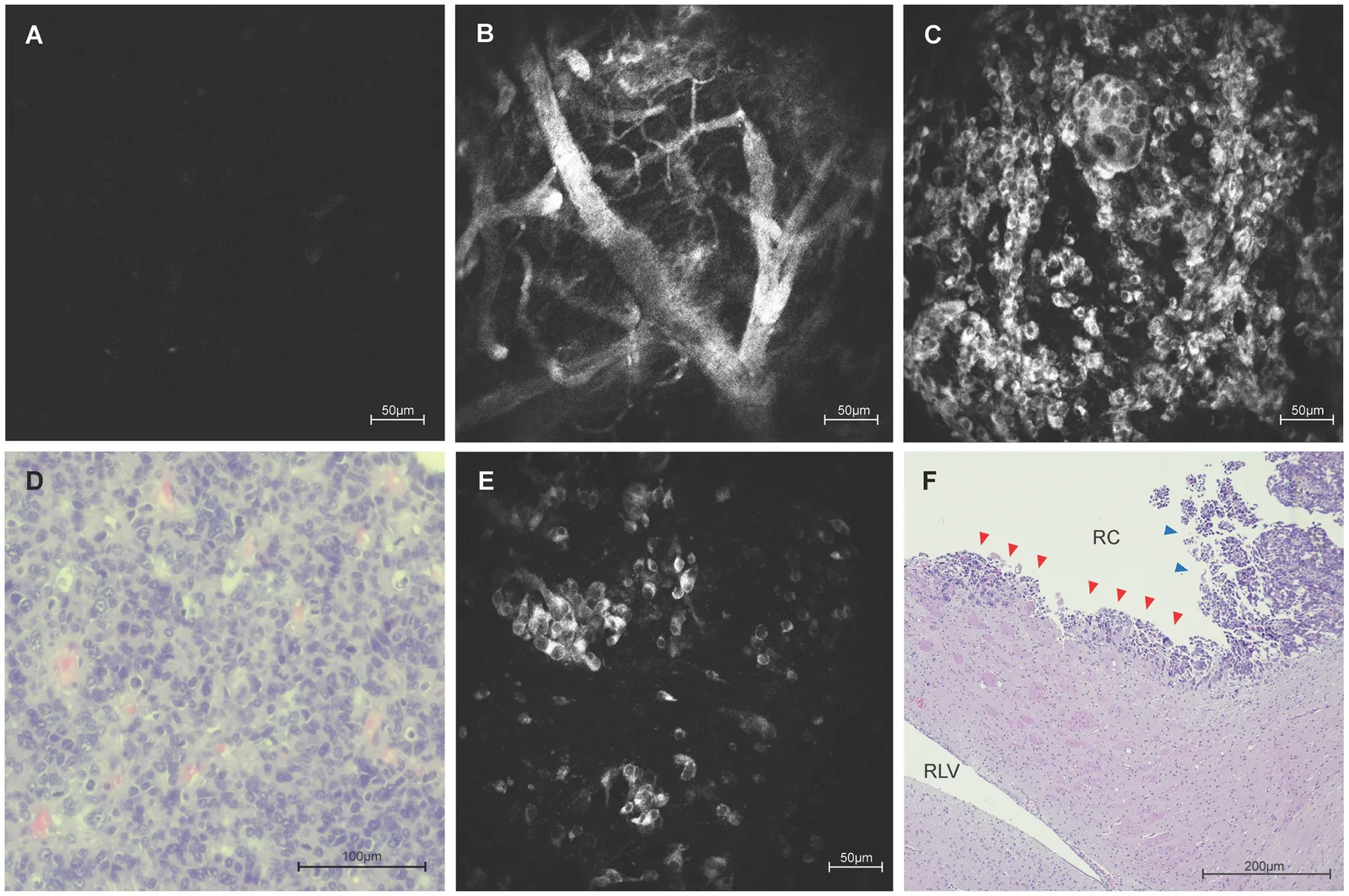
Confocal laser endomicroscopy (CLE) imaging using intravenous indocyanine green. **(A)** CLE imaging of the contralateral hemisphere showed no significant fluorescence signal, except for weak spots of fluorescence. **(B)** Cortical vessels of various calibers can be seen with flowing intraluminal erythrocytes as dark streaks. **(C)** CLE imaging of tumor tissue revealed clustered, heterogeneous cells with fluorescent cytoplasm and nonfluorescent nuclei, consistent with the tumor morphology seen in **(D)** hematoxylin and eosin (H&E)-stained sections. **(E)** CLE imaging and **(F)** H&E-stained sections show scattered tumor cells and tumor cell nests at the medial (*red arrowheads*) and inferior (*blue arrowheads*) tumor margins. RC, resection cavity; RLV, right lateral ventricle. Used with permission from Barrow Neurological Institute, Phoenix, Arizona.

NIR CLE imaging was started immediately after intravenous ICG injection. ICG fluorescence signal was detected within the first few minutes. Minimal fluorescence signal was detected when imaging the contralateral hemisphere cortex (**Figure 1A**). Weak fluorescence spots were observed in some areas, but not consistently across all ROIs. Cortical blood vessels of various sizes were noted (**Figure 1B**). Within blood vessels, streams of nonfluorescent erythrocytes were identified.

Within tumor tissue, ICG fluorescence revealed densely populated structures of strong fluorescence surrounding dark, round areas with no signal (**Figures 1C, D**). This distribution pattern is consistent with the localization of ICG in cytoplasm (6, 14). The cells appear in different sizes and shape, and occasional mitotic figures are observed. The extracellular background had minimal to no fluorescence signal. After careful tumor removal using the surgical microscope, the probe was again positioned in contact with the resection bed. Multiple ROIs within the resection bed were imaged and marked for histology assessment. Clusters of pleomorphic tumor cells were identified consistently at tumor margins and confirmed by H&E-stained histology (**Figures 1E, F**).

**Figure 2:**
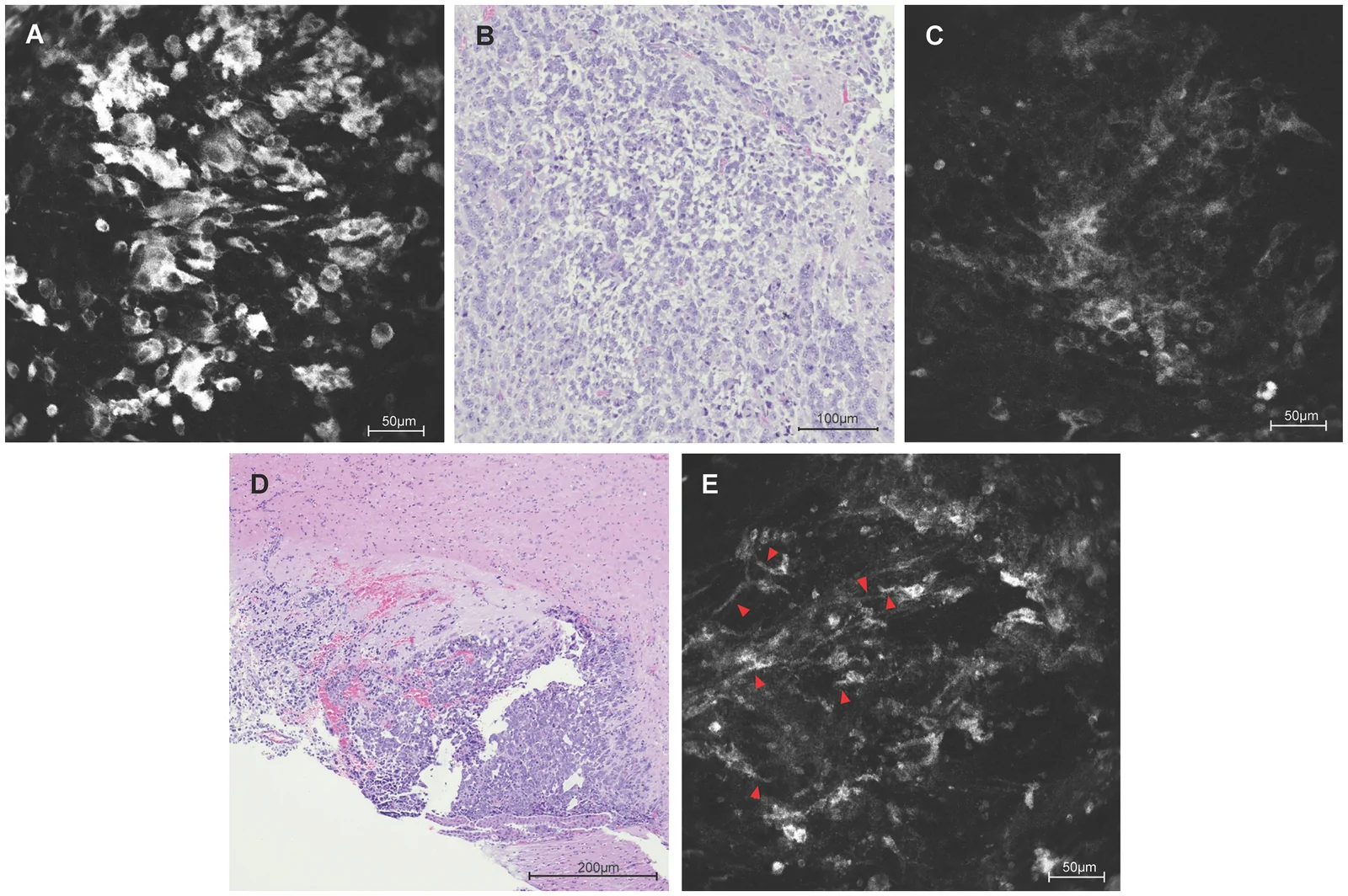
Confocal laser endomicroscopy (CLE) imaging using topical indocyanine green (ICG). **(A)** CLE imaging of tumor tissue after topical application of ICG produced images similar to those obtained with intravenous ICG, showing cells with fluorescent cytoplasm and nonfluorescent nuclei, consistent with the tumor morphology seen in **(B)** hematoxylin & eosin (H&E)-stained sections. **(C)** At the resection margins, topical ICG staining revealed islets of remaining tumor cells consistent with **(D)** H&E-stained sections, despite an extracellular background with slightly more fluorescence. **(E)** Topical ICG applied to the contralateral hemisphere stained sparse cells with a morphology that was distinct from tumor cells. These cells had smaller nuclei and prominent processes (*red arrowheads*), giving the extracellular background a mesh-like appearance. Used with permission from Barrow Neurological Institute, Phoenix, Arizona.

CLE imaging of the tumor using topical ICG produced images like those with intravenous ICG (**Figures 2A, B**). At the resection margins, topical ICG staining also revealed islets of remaining tumor cells, despite an extracellular background with slightly more fluorescence (**Figures 2C, D**). When imaging the contralateral hemisphere, topical ICG stained sparse cells with morphology distinct from tumor cells (**Figure 2E**). These cells have smaller nuclei and prominent processes, giving the extracellular background a mesh-like appearance.

### 3.3 Imaging with blue laser CLE and FNa

**Figure 3:**
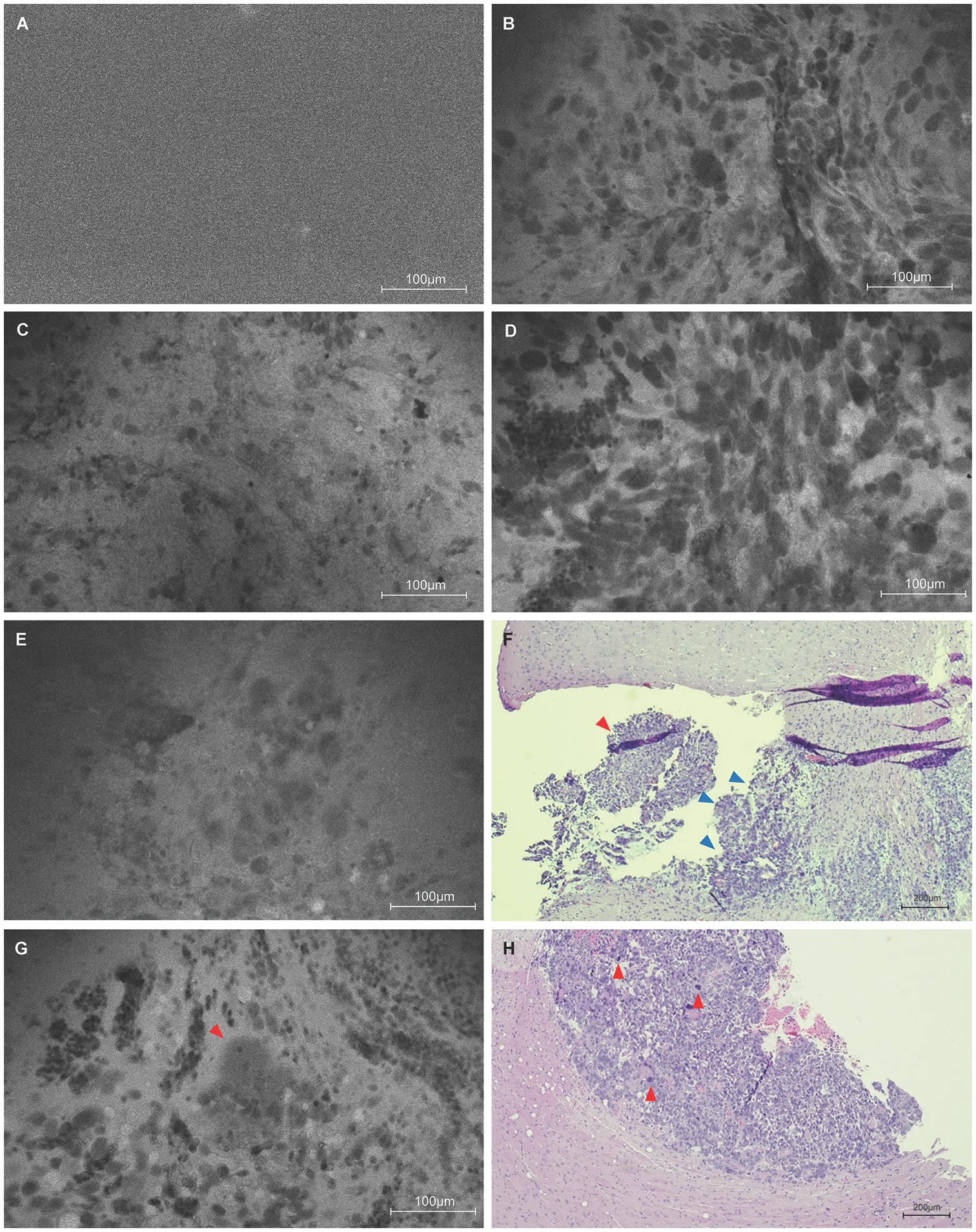
Confocal laser endomicroscopy (CLE) imaging using systemic and topical fluorescein sodium (FNa). **(A)** CLE imaging of the contralateral hemisphere cortex after application of systemic (intraperitoneal) FNa administration showed no significant fluorescence signal because the uptake of FNa by normal brain tissue was blocked by the intact blood-brain barrier. **(B)** CLE imaging after application of systemic FNa to the tumor. In tumor tissue, systemic FNa extravasated and accumulated in the extracellular matrix, allowing large, pleomorphic tumor cells to be seen. **(C)** FNa applied topically to normal brain tissue showed small, dark cells with much lower density and heterogeneity than tumor cells. **(D)** FNa applied topically to tumor tissue showed a pattern similar to that of CLE imaging with systemic FNa. **(E)** CLE image using systemically applied FNa revealed clustered tumor cells. **(F)** Corresponding image from hematoxylin and eosin (H&E)-stained section shows tumor tissue within the resection cavity (*red arrowhead*) and in the inferior resection bed (*blue arrowheads*). **(G)** CLE image at the resection margin after application of topical FNa similarly showed large pleomorphic tumor cells (*red arrowhead*). **(H)** Corresponding image from H&E-stained section shows large, atypical cells (*red arrowheads*). Used with permission from Barrow Neurological Institute, Phoenix, Arizona.

Features observed in CLE images with systemic FNa administration match what were reported (5, 11, 22). No fluorescence was detected from the contralateral hemisphere cortex because the uptake of FNa by normal brain tissue was blocked by the BBB (**Figure 3A**). Lack of a BBB in the tumor led to extravasation of FNa that accumulated in the extracellular matrix. Large, pleomorphic tumor cells were shown as dark, round silhouettes of varying sizes and shapes (**Figure 3B**). Staining normal brain tissue with topical FNa showed some dark, small cells with much less density and heterogeneity compared to the tumor cells (**Figures 3C, D**). At the resection margins, both systemic and topical FNa uncovered large pleomorphic tumor cells (**Figures 3E–H**).

### 3.4 Signal-to-background ratio analysis

**Figure 4:**
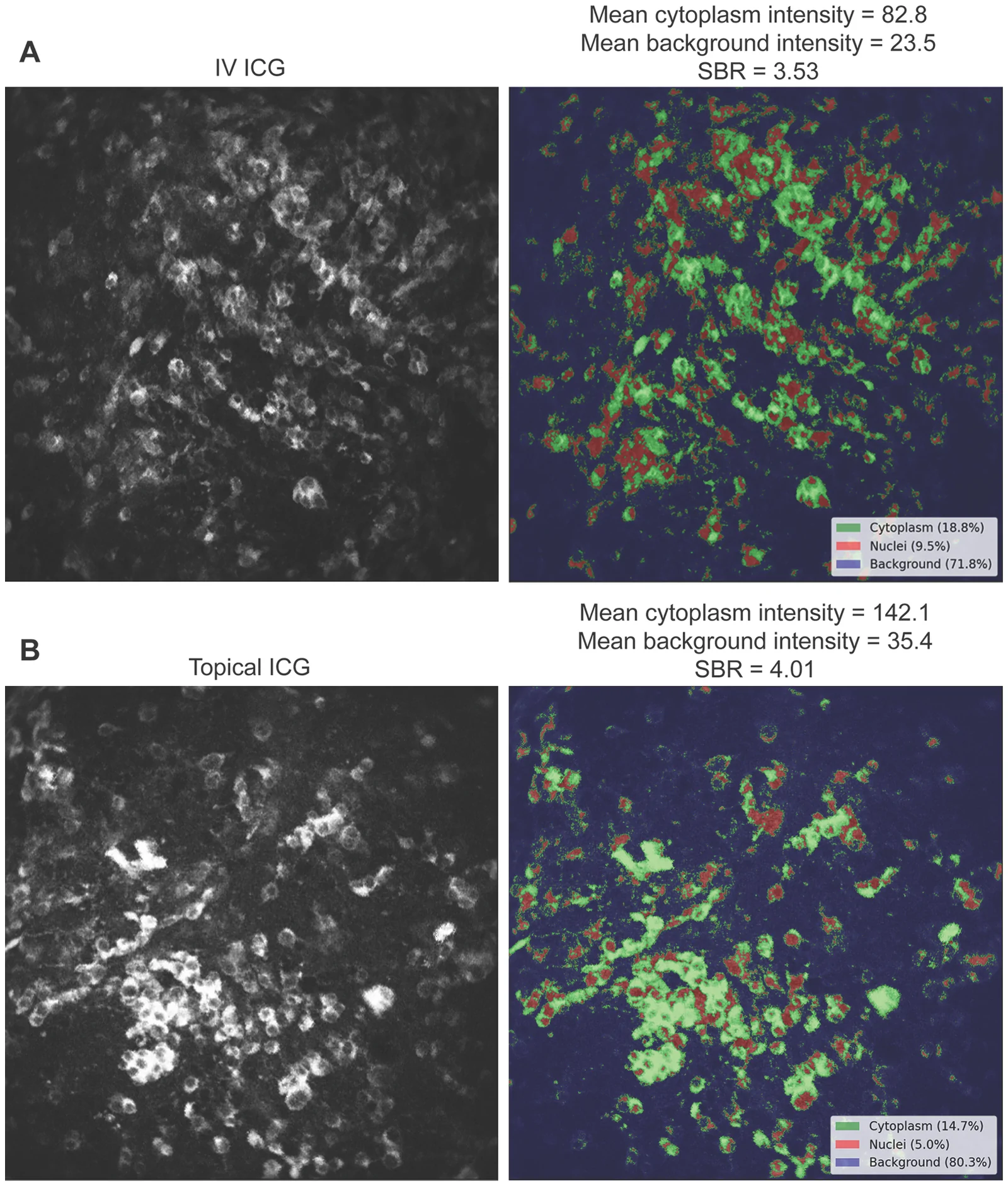
Signal-to-background ratio (SBR) analysis of confocal laser endomicroscopy (CLE) imaging of the tumor after application of intravenous (IV) versus topical indocyanine green (ICG). **(A)** Representative CLE image of tumor using IV ICG (*left*) and corresponding segmentation map (*right*). Mean cytoplasm intensity = 82.8, mean background intensity = 23.5, SBR = 3.53. **(B)** Representative CLE image of tumor after topical application of ICG (*left*) and corresponding segmentation map (*right*). Mean cytoplasm intensity = 142.1, mean background intensity = 35.4, SBR = 4.01. Overall, topical ICG produced higher cytoplasmic fluorescence intensity and a higher SBR compared to IV ICG. Used with permission from Barrow Neurological Institute, Phoenix, Arizona.

SBR analysis was performed on CLE images of tumor tissue acquired with intravenous and topical ICG using the NIR CLE system, with 132 SBR measurements obtained from intravenous ICG images and 174 from topical ICG images. Topical ICG produced significantly higher cytoplasmic fluorescence intensity than intravenous ICG (median 105.0 vs. 93.8, p < 0.0001), while background fluorescence intensity did not differ significantly between the two administration routes (median 24.3 vs. 25.9, p = 0.425). The median SBR of CLE images with intravenous ICG and topical ICG were 4.9 (IQR 4.0-5.8) and 5.7 (IQR 4.2-6.7) (**Figure 4**). A linear mixed-effects model using all images showed a fold-change of 1.16 (95% CI 0.90–1.50, p = 0.26) for topical ICG compared with intravenous SBR, directionally consistent with the image-level finding but not statistically significant. Thus, topical ICG application produced cell-to-background contrast noninferior to intravenous ICG while maintaining the same cytoplasmic staining pattern.

## 4 Discussion

As the first developed and clinically approved CLE imaging system, the blue laser FNa-based CLE system has been the most studied and reported. Although previous studies have demonstrated that CLE imaging of brain tumors with FNa yields diagnostic accuracy comparable to frozen section histological analysis, with reported accuracy rates exceeding 90% in some series (4, 23, 24), the lack of cellular uptake and specific staining profile make it less reliable when imaging tissue with more ambiguous histoarchitecture such as low-grade tumors and at tumor margins, where sensitivity, i.e., tumor detection, significantly decreases (25, 26). FNa accumulates in the extracellular matrix of tissue with BBB disruption rather than undergoing tumor cell-specific uptake (10, 11, 27). Although this pattern is diagnostically useful and has been validated in clinical studies (2, 4, 24, 28, 29), it inherently limits the ability to assess individual cell morphology and makes the presence of erythrocytes an almost inevitable diagnostic challenge.

**Figure 5:**
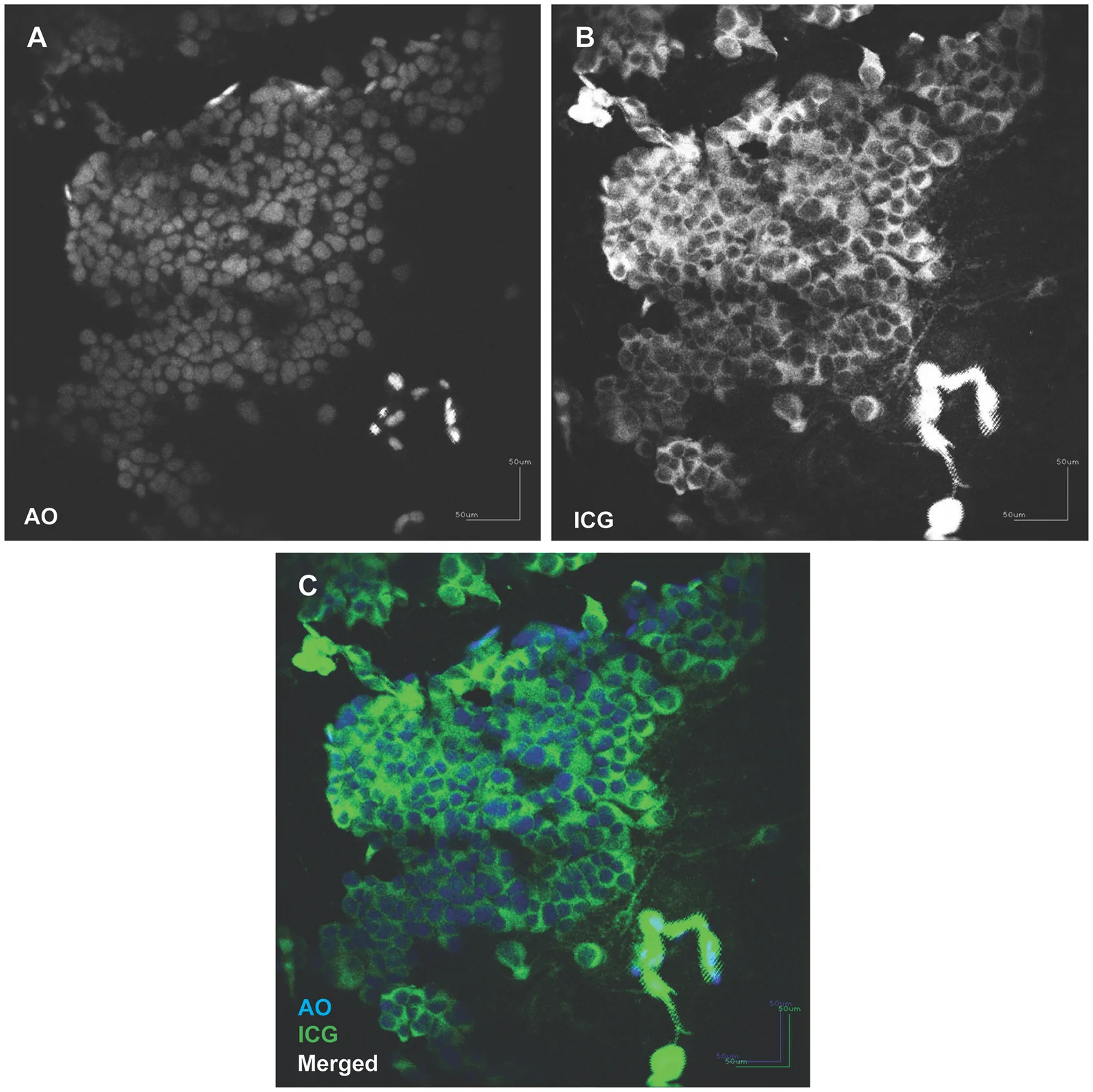
Dual-channel confocal laser endomicroscopy (CLE) imaging of tumor tissue after application of acridine orange (AO) and indocyanine green (ICG). **(A)** Blue laser channel (488 nm) showing tumor cell nuclei stained with AO. **(B)** Near-infrared channel (785 nm) showing tumor cells with cytoplasm stained with ICG. **(C)** Merged image demonstrating the complementary staining patterns of AO and ICG. Used with permission from Barrow Neurological Institute, Phoenix, Arizona.

With ICG, the cytoplasm of tumor cells exhibited prominent fluorescence, while the nuclei remained dark, enabling visualization of both nuclear size and shape and overall cell morphology. This was clearly confirmed by imaging tumor tissue with cCeLL’s two laser channels (775 nm and 488 nm) in tandem without changing or repositioning the probe. After simultaneous topical application of ICG and acridine orange, a blue laser-excitable fluorophore that selectively stains cell nuclei, ICG fluorescence localized to the cytoplasm while acridine orange labeled the nuclei, with the merged image demonstrating distinct, non-overlapping subcellular localization of both fluorophores (**Figure 5**). This positive-contrast imaging enabled clear assessment of cell size, nuclear-to-cytoplasmic ratio, and clear differentiation between tumor consisting of densely packed, large, pleomorphic cells and normal brain, features that were difficult to appreciate with FNa-based imaging. The mechanism of ICG intracellular uptake is thought to involve cell membrane transporters and endocytosis mechanisms, with ICG accumulating in the cytoplasm but not in the nucleus, and with intracellular retention persisting significantly longer in tumor cells than in normal tissue (30–32). In addition to its intracellular localization, NIR excitation and emission wavelengths fall within the optical tissue transparency window, affording deeper tissue penetration and reduced background autofluorescence (33–35). This reduction in background autofluorescence is in agreement with our observation that no significant background autofluorescence was seen in the NIR CLE images from the tumor tissue and contralateral normal hemisphere. Tissue autofluorescence is observed with blue-laser CLE systems in brain tumor and nontumor tissue with and without the use of FNa, although its correlation with certain pathology types is inconsistent, and its diagnostic value is not well established (4, 36).

*In vivo* NIR CLE imaging of brain tumors, using a preclinical system with intravenous ICG, was demonstrated by Martirosyan et al. in 2011 (14). NIR CLE with ICG, an intracellular fluorescent contrast agent, detected individual tumor cells and satellite tumor clusters within peritumoral tissue, delineated a definitive tumor border, and revealed fluorescent microvascular and subcellular structures, correlating well with standard histological features. ICG fluorescence could be detected in tumor tissue immediately after injection, fully saturating the tissue 15 minutes later, and persisted for over 1 hour with continuous CLE imaging.

cCeLL uses resonant scanning with a Lissajous scanning pattern instead of traditional point-by-point raster scanning to achieve a significantly faster image acquisition speed of 10 frames per second at the expense of a slight but noticeable reduction in image quality. The faster image acquisition speed significantly reduces motion artifacts commonly seen with blue-laser CLE systems and allows users to quickly scan tissue with the probe. The scanning pattern may be visible in some frames, leading to pixelated or completely distorted images. However, the effect on overall imaging appears to be minor, given the high number of unaffected images. Hong and colleagues first demonstrated the clinical feasibility of the cCeLL NIR CLE system imaging excised tumor tissue (6). In the subsequent multicenter pilot controlled ex vivo imaging study by Byun et al. (15), the cCeLL system achieved a diagnostic accuracy of 89.2% compared with 86.5% for frozen section analysis, with both methods showing equivalent sensitivity of 92.2%, supporting the diagnostic potential of ICG-based NIR CLE (15). Although CLE was originally conceived as an intraoperative optical biopsy tool intended for neuropathologist interpretation, increasing evidence indicates that neurosurgeons who operate the imaging system can acquire comparable proficiency in interpreting CLE images (26).

ICG is routinely given intravenously for intraoperative vascular imaging. Intraoperative wide-field fluorescence imaging of brain tumors has also been investigated with intravenous ICG, administered either as a single standard dose (37, 38) or a high-dose for delayed second-window imaging (13, 39, 40), for its propensity to accumulate in brain tumor tissue with damaged BBB. In our study, *in vivo* CLE imaging with topical ICG produced staining pattern identical to intravenous ICG in tumor tissue. At the image level, topical ICG staining also showed a higher SBR than intravenous ICG. However, this difference did not reach statistical significance once the clustering of images within animals was accounted for. Quantitative analysis of the cytoplasm and background intensity data revealed that the higher SBR observed with topical ICG was driven specifically by increased cytoplasmic fluorescence intensity, while background fluorescence intensity did not differ significantly. This effect may be due to topical application delivering ICG directly to exposed tissue at higher local concentrations, resulting in greater intracellular uptake. In our experience, topically applied ICG solution with a concentration as low as 0.0001% produced robust fluorescence for CLE imaging. It is important to rinse the tissue thoroughly to remove the excess ICG solution that may accumulate in microscopic pockets in the tissue and cause oversaturation of cell and background signal in the CLE images.

**Figure 6:**
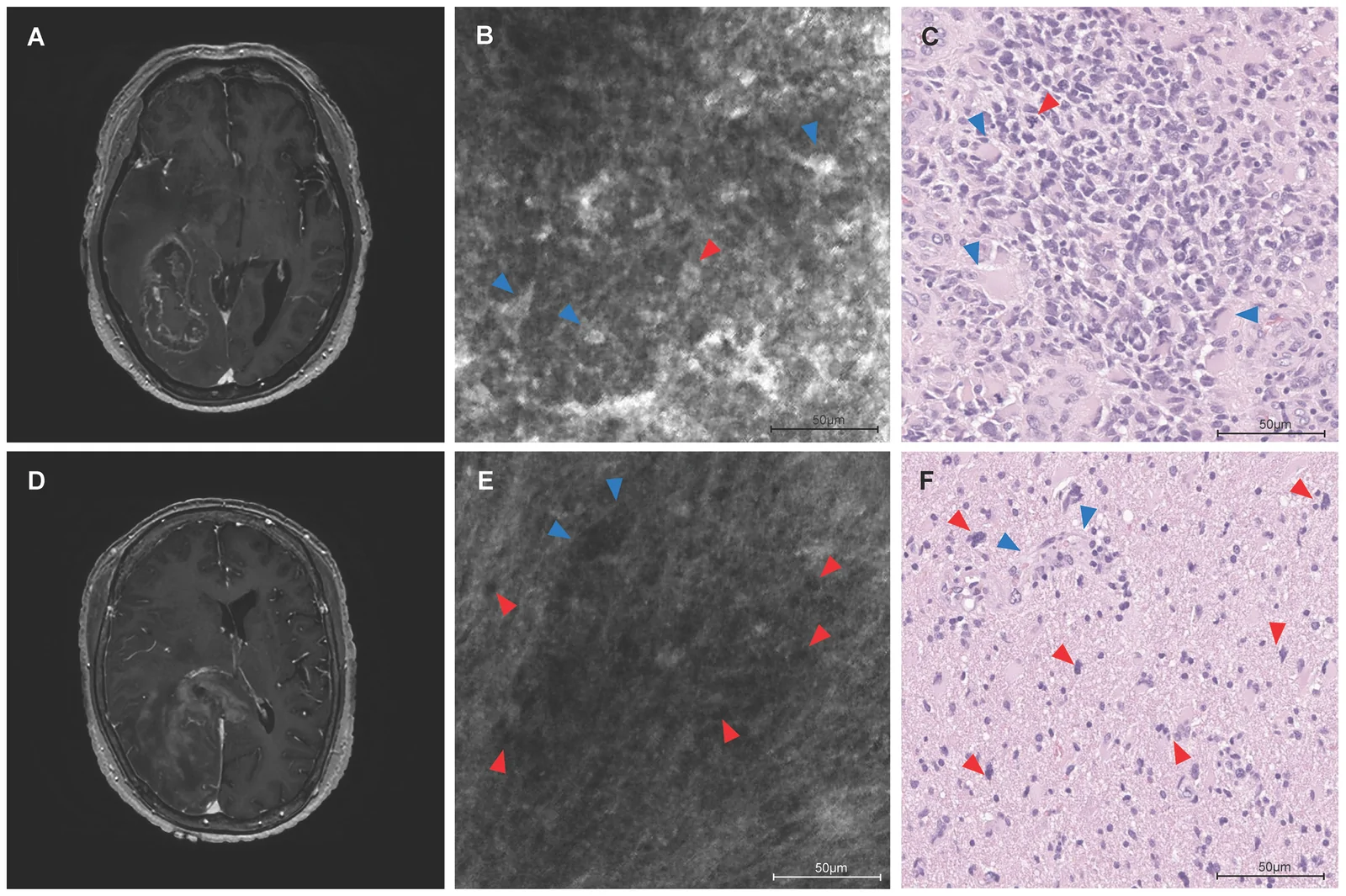
*In vivo* near-infrared confocal laser endomicroscopy (CLE) imaging with topical ICG application in two human glioblastoma cases. *Case 1.* **(A)** Preoperative contrast-enhanced T1-weighted MRI showed an enhancing lesion in the right temporoparietal lobe with significant necrosis and perilesional edema. **(B)** CLE image of tumor tissue from the same patient demonstrating a hypercellular area composed of numerous dark, round-to-oval tumor nuclei. Scattered gemistocytic tumor cells with abundant bright cytoplasm are present (*blue arrowheads*). A mitotic-like dark structure is visible within a bright cytoplasmic area (*red arrowhead*). **(C)** Corresponding hematoxylin and eosin (H&E)-stained section of the same lesion showing a densely cellular tumor with hyperchromatic nuclei and scattered gemistocytic tumor cells with abundant eosinophilic cytoplasm (*blue arrowheads*). A mitotic figure can be seen (*red arrowhead*). *Case 2.* **(D)** Preoperative contrast-enhanced T1-weighted MRI shows a large heterogeneously enhancing mass lesion that involves the right parieto-temporo-occipital lobe with extension to the basal ganglia and the splenium of the corpus callosum with parenchymal edema. **(E)** CLE image of the same tumor demonstrating numerous dark, round-to-oval tumor nuclei (*red arrowheads*) within a fibrillary neuropil background. With topical ICG, blood vessels appear as elongated dark structures (*blue arrowheads*). **(F)** Corresponding H&E-stained section of the same lesion showing scattered hyperchromatic tumor nuclei (*red arrowheads*) embedded in neuropil. A blood vessel can be seen *(blue arrowheads).* Used with permission from Seoul National University Hospital and Korea University Anam Hospital.

When the fluorescence is excessively intense, the laser power and brightness settings in the CLE system can be reduced to maintain appropriate contrast. Our data showed that with a proper topical staining protocol and adjustment of laser power and brightness settings, topical ICG increases cytoplasmic fluorescence intensity without proportionally increasing the background signal, yielding a higher SBR and better contrast. This pattern was also observed in early stage clinical *in vivo* NIR CLE imaging with topical ICG (**Figure 6**), although improvement in imaging protocol and systematic validation is needed to optimize *in vivo* imaging in the more complicated intraoperative setting.

We demonstrated that, when applied topically, ICG fluorescence was reliably detected within tumor and, importantly, normal brain cells. Although topically applied ICG stains tumor and normal tissue indiscriminately and loses the tumor selectivity seen in operative microscope wide-field imaging with systemic ICG, its cytoplasmic localization allows for differentiation between normal and tumor tissue based on cellular morphology. Topical ICG applied to the contralateral normal cortical surface clearly distinguished tumor from normal histoarchitecture. Stained cells were smaller, with smaller nuclei and prominent cellular processes, consistent with morphology of astrocytes. Background fluorescence was more prominent with mesh-like appearance from the cellular processes. Visualizing normal brain cytoarchitecture with topical ICG, while maintaining clear morphological distinction from tumor cells, is a valuable and convenient feature for intraoperative margin assessment. Similarly, topical ICG may have the unique advantage when used for imaging low-grade, nonenhancing gliomas. By bypassing the less disrupted BBB that normally prevents the extravasation of the fluorophores, topical application can deliver ICG to tissue that is normally not stained when the fluorophore is given systematically. This finding needs to be confirmed in future human studies and can potentially be impactful in intraoperative *in vivo* imaging of nonenhancing brain tumors.

For FNa, the systemic and topical routes showed markedly different behaviors. Systemic FNa did not produce detectable fluorescence signal at the resection bed or contralateral brain, consistent with the known BBB-dependent mechanism of FNa distribution (10, 11, 41). Topical FNa showed cells at tumor, margin, and normal brain sites with a similar appearance, producing cells that appeared as dark round structures that only differ in size and density. This limits its usability for imaging lower-grade tumors or recurrent, previously treated tumors, in which the sizes of neoplastic and nonneoplastic cells typically do not differ significantly. FNa-based CLE shows higher sensitivity compared to the more selective wide-field 5-ALA fluorescence under operating microscopy for detecting tumor cells at malignant glioma margin areas or in low-grade or nonenhancing gliomas (42), although there are currently no clinically approved CLE systems for imaging protoporphyrin IX fluorescence.

Besides FNa and ICG, several other fluorophores with specific staining profiles have been investigated for imaging brain tumors. Visualized with benchtop confocal microscopy, topically applied acridine orange, acriflavine, and cresyl violet each provided rapid fluorescent labeling with characteristic subcellular staining selectivity in surgical resected brain tumor specimens (43). Acridine orange intercalates into nucleic acids and preferentially labels cell nuclei, producing images with prominent nuclear fluorescence that enable assessment of nuclear density, pleomorphism, and nuclear-to-cytoplasmic ratio (22, 43). Acriflavine similarly labels nuclei but also highlights extracellular matrix and connective tissue elements, whereas cresyl violet stains cytoplasm and cell membrane with weak fluorescence (9, 43). Although these fluorophores are used with CLE imaging in other body regions, they are not currently used in the brain because acridine compounds are associated with mutagenicity (44, 45) and cresyl violet lacks regulatory approval, thereby underscoring the clinical advantage of ICG as a selective fluorophore that is already approved and routinely used in neurosurgery.

Although the tumor is aggressively expansive, tumor borders in the syngeneic GL261 model are better demarcated than those seen in human gliomas, which may overestimate CLE’s ability to distinguish tumor from normal brain at infiltrative margins. The syngeneic immunocompetency of the tumor line has been established as an appropriate translational imaging study substrate. This study did not include healthy, non-tumor-bearing control animals, and baseline tissue autofluorescence was not independently quantified. Considering that the diagnostic value of autofluorescence in CLE imaging is not clearly established, such omission does not undermine our observations and is not practically relevant to the local surgical application of CLE. Although SBR was measured and compared between intravenous and topical ICG staining, it was restricted to images of tumor tissue because normal brain tissue showed minimal uptake of intravenous ICG. The automated frame sampling and SBR measurement workflow was designed to minimize bias from manual segmentation and facilitate rapid measurement of hundreds of images, but it may be affected by slight motion artifact and scanning pattern. CLE image interpretation and H&E histopathological assessment were each performed by a single reviewer who was not blinded. Future studies assessing diagnostic performance should incorporate blinded, multirater assessment, with formal interrater agreement statistics.

Future directions include several important avenues. Systematic clinical validation in a dedicated clinical cohort with correlation to histopathology may be necessary before conclusions based on the murine model can be translated to patients. Development and validation of tumor-specific fluorescent probes combining the NIR spectrum of ICG with molecular targeting of tumor-specific markers could further improve the specificity of CLE-based tumor identification (46, 47). Advances in artificial intelligence and deep learning for automated CLE image interpretation have shown promise in automated, accurate interpretation of images and in reducing the learning curve associated with CLE image analysis (48, 49). Multimodal imaging approaches that combine wide-field second-window ICG fluorescence-guided surgery with NIR CLE imaging could provide both macroscopic tumor visualization and microscopic, cellular-resolution optical biopsy.

## 5 Conclusion

ICG-based NIR CLE imaging provides high-quality morphological detail of brain tumor cells in a GL261 mouse glioma model. ICG produced a distinctive intracellular fluorescence pattern with prominent cytoplasmic staining and dark nuclei, enabling direct visualization of cell size and nuclear morphology and clear differentiation between tumor and normal brain tissue. By contrast, FNa produced the characteristic extracellular fluorescence pattern, providing useful yet less detailed morphological information. Topical ICG maintained the same staining pattern as intravenous ICG with a similar SBR. These findings support ICG as a valuable fluorophore for CLE optical biopsy of brain tumors and provide a foundation for future clinical translation of NIR CLE imaging approaches to improve intraoperative cellular resolution diagnosis and guidance during brain tumor surgery.

## Data Availability

The original contributions presented in the study are included in the article/supplementary material, and further inquiries can be directed to the corresponding author.
